# Utility of rabies neutralizing antibody detection in cerebrospinal fluid and serum for ante-mortem diagnosis of human rabies

**DOI:** 10.1371/journal.pntd.0007128

**Published:** 2019-01-29

**Authors:** Tina Damodar, Reeta S. Mani, P. V. Prathyusha

**Affiliations:** 1 Department of Neurovirology, WHO Collaborating Centre for Reference and Research in Rabies, National Institute of Mental Health and Neurosciences (NIMHANS), Bangalore, India; 2 Department of Biostatistics, National Institute of Mental Health and Neurosciences (NIMHANS), Bangalore, India; Wistar Institute, UNITED STATES

## Abstract

**Background:**

Early ante-mortem laboratory confirmation of human rabies is essential to aid patient management and institute public health measures. Few studies have highlighted the diagnostic value of antibody detection in CSF/serum in rabies, and its utility is usually undermined owing to the late seroconversion and short survival in infected patients. This study was undertaken to examine the ante-mortem diagnostic utility and prognostic value of antibody detection by rapid fluorescent focus inhibition test (RFFIT) in cerebrospinal fluid (CSF)/serum samples received from clinically suspected human rabies cases from January 2015 to December 2017.

**Methodology/Principal findings:**

Samples collected ante-mortem and post-mortem from 130 and 6 patients with clinically suspected rabies respectively, were received in the laboratory during the study period. Ante-mortem laboratory confirmation was achieved in 55/130 (42.3%) cases. Real time PCR for detection of viral nucleic acid performed on saliva, nuchal skin, brain tissue and CSF samples could confirm the diagnosis in 15 (27.2%) of the 55 laboratory confirmed cases. Ante-mortem diagnosis could be achieved by RFFIT (in CSF and/or serum) in 45 (34.6%) of the 130 clinically suspected cases, accounting for 81.8% of the total 55 laboratory confirmed cases. The sensitivity of CSF RFFIT increased with the day of sample collection (post-onset of symptoms) and was found to be 100% after 12 days of illness. Patients who had received prior vaccination had an increased probability of a positive RFFIT and negative PCR result. Patients who were positive by RFFIT alone at initial diagnosis had longer survival (albeit with neurological sequelae) than patients who were positive by PCR alone or both RFFIT and PCR.

**Conclusions/Significance:**

Detection of antibodies in the CSF/serum is a valuable ante-mortem diagnostic tool in human rabies, especially in patients who survive beyond a week. It was also found to have a limited role as a prognostic marker to predict outcomes in patients.

## Author summary

Ante-mortem diagnosis of human rabies is essential for patient management and public health measures. The detection of virus specific antibodies in the CSF/serum of patients with suspected rabies is thought to have a limited diagnostic role owing to late seroconversion and short survival in rabies. We examined the diagnostic and prognostic utility of antibody detection by rapid fluorescent focus inhibition test (RFFIT) in CSF/serum samples received from clinically suspected human rabies cases (2015–2017). RFFIT (in CSF and/or serum) could confirm ante-mortem diagnosis in 45 (34.6%) of the 130 clinically suspected cases, accounting for 81.8% of the total 55 laboratory confirmed cases. The sensitivity of CSF RFFIT increased with the day of sample collection (post-onset of symptoms) and was found to be 100% after 12 days of illness. Patients who had received prior vaccination had an increased likelihood of a positive RFFIT and negative PCR result. Patients who were positive by RFFIT alone at initial diagnosis had longer duration of survival, although with poor functional outcomes. Antibody detection by RFFIT in CSF/serum was found to have a diagnostic utility especially in patients who survived beyond a week and a limited prognostic role in human rabies.

## Introduction

Rabies is an acute progressive, fatal encephalomyelitis caused by viruses of the Lyssavirus genus (Order *Mononegavirales*, Family *Rhabdoviridae*). *Rabies lyssavirus* (RABV), the prototype virus of the Lyssavirus genus is the most common causative agent of rabies, usually transmitted through the bite of an infected mammal. Dog-transmitted rabies accounts for most of the human cases reported worldwide. This zoonotic disease causes an estimated 61,000 human global deaths annually, mostly in Asia and Africa; India accounts for a third of the global disease burden [1, 2].

Two classical forms of rabies are generally recognized: furious (or encephalitic) and paralytic. However, a diagnosis of rabies based solely on clinical features, especially in the absence of history of exposure is difficult and often unreliable [1]. Indeed, the clinical presentation is often variable and may actually represent a continuum of signs and symptoms [3]. Laboratory confirmation must therefore be done in all suspected cases wherever feasible- to rule out clinical mimics, aid patient management, avoid unwarranted medical tests and treatment, and also help in case closure and grief counselling with family members.

The gold standard for rabies confirmation is demonstration of viral antigen by fluorescent antibody test (FAT) on brain tissue obtained post-mortem. However, obtaining brain tissue after death remains a challenge and is rarely performed due to religious, logistic or bio-safety related reasons [4]. Despite significant developments in laboratory techniques, ante-mortem diagnosis of human rabies is fraught with several challenges. While a positive validated result is indicative of rabies, a negative result does not essentially rule out a diagnosis of rabies in all cases, which is a major limitation of ante-mortem testing [1]. Therefore, a combination of several tests on multiple clinical samples, with serial sampling whenever feasible is recommended to increase the sensitivity of ante-mortem diagnosis [5, 6].

Nucleic acid amplification techniques like PCR on clinical samples like saliva, cerebrospinal fluid (CSF), nuchal skin, extracted hair follicles and urine are increasingly being used for ante-mortem diagnosis of rabies [4–10]. Testing for virus specific antibodies in serum and CSF is considered to be of limited value since seroconversion occurs late in the course of the disease [11] and many patients succumb to the infection within a few days of onset of symptoms. However, the detection of virus-specific antibodies in the serum of unvaccinated individuals, or in the CSF of both vaccinated and unvaccinated individuals, can be valuable in diagnosis, especially in cases where survival is prolonged beyond a week [5].

Clinical samples from suspected human rabies cases from various hospitals in India are received at the Neurovirology laboratory, NIMHANS, Bangalore for diagnostic testing. A retrospective analysis was undertaken to examine the ante-mortem diagnostic utility and prognostic value of antibody detection in CSF/serum samples received from clinically suspected human rabies cases from January 2015 to December 2017.

## Methods

### Ethics statement

All human clinical specimens included in this study were received at the Neurovirology laboratory for diagnostic confirmation of rabies in clinically suspected cases as requested by the attending physicians of the patients. None of the samples were obtained from infected patients specifically for this study. All human samples used in the study were anonymized. Considering the retrospective nature of the study, approval from the participating hospitals and the institutional review board was not required.

### Clinical samples

Clinical samples from 136 patients with suspected human rabies from various hospitals across the country were received during the study period. The clinical samples included CSF, blood (serum), saliva, nuchal skin and brain biopsy collected ante-mortem, and brain tissue obtained post-mortem. The referring physicians were instructed to complete a standardized case history form with demographic and clinical details, including history of animal exposure and details of post-exposure management for all patients for whom rabies diagnostic testing was requested.

### Laboratory methods

Laboratory tests were performed as requested by the referring physicians. Nucleic acid extraction followed by real-time TaqMan PCR was performed as described earlier [4] on CSF, saliva, nuchal skin biopsy and brain tissue samples, for detection of RABV RNA. Brain tissues were also subjected to the Fluorescent Antibody Test (FAT) for the detection of RABV nucleoprotein antigen [12].

CSF and serum samples were tested for rabies virus neutralising antibody (RVNA) titres by rapid fluorescent focus inhibition test (RFFIT), using a WHO recommended procedure [13] with some modifications as described earlier [14]. The antibody titers were expressed in International units (IU/ml) in comparison to an in house reference serum calibrated against 2nd International reference serum obtained from National Institute of Biological Standards, UK. The lower limit of detection of the assay was 0.1 IU/ml. Any detectable titre in a single CSF sample (irrespective of prior vaccination status) or serum sample (in patients with no prior vaccination), was considered positive. In patients who had received prior rabies vaccination (partial or complete), or where history of prior vaccination was unknown, only paired sera demonstrating a four-fold or greater rise in antibody titres was considered positive. A clinically suspected case which was positive by one or more of the laboratory tests above was considered a laboratory confirmed case of rabies [1].

### Statistical methods

Statistical analysis was carried out using IBM-SPSS version 22 for windows. The qualitative variables were described using frequencies and percentages; median and interquartile range was used to describe the quantitative variables. Kruskal Wallis test was used to compare the titres among different groups. Spearman’s correlation coefficient was used to determine the correlation between day of sample collection (post-onset of symptoms) and the RFFIT titres. Fisher’s exact test was used to find the association between the laboratory test outcome and the vaccination status.

## Results

Clinical samples from 136 patients with suspected rabies were received in the Neurovirology laboratory for diagnostic testing from January 2015 to December 2017. The age of patients ranged from 1 to 78 years (mean 29.6 years) and 91(66.9%) were male. Children up to 12 years of age accounted for 42/136 (30.8%) of the cases. History of animal exposure was known in 130/136 (95.5%) of cases. 129 (94.8%) patients had exposure (bites, scratches) to dogs and one patient reported mongoose bite.

### Laboratory tests

Samples collected ante-mortem and post-mortem were received from 130 and 6 patients with clinically suspected rabies respectively; laboratory confirmation could be achieved in 61/136 (44.8%) cases. Ante-mortem diagnosis was achieved in 55/130 (42.3%) cases (Table 1).

**Table 1:** Ante-mortem diagnosis of rabies in clinically suspected cases (n = 130).

Laboratory Test	Number of cases (%)
Laboratory confirmed in	55 (42.3%)
Positive by RFFIT (CSF and/or Serum)	45 (81.8%)
Positive by RFFIT alone(CSF and/or Serum)	40 (72.7%)
Positive by CSF RFFIT only	21 (38.2%)
Positive by Serum RFFIT only	2 (3.6%)
Positive by CSF & Serum RFFIT	17 (30.9%)
Positive by both PCR & RFFIT (CSF and/or Serum)	5 (9%)
Positive by PCR alone (in single or multiple samples)	10 (18.1%)

### Real time PCR

Real time PCR was performed on CSF, saliva, nuchal skin and brain biopsy samples. 4/88 (4.5%) CSF samples, 10/63 (15.8%) saliva samples and 5/28 (17.8%) nuchal skin biopsies tested were positive for rabies viral RNA. Ante-mortem brain biopsy was received from only one patient and it was positive for rabies by FAT and PCR. A total of 20 samples from 15 patients were positive by PCR [only saliva (6); only nuchal skin (3); only CSF (1); Brain (1); saliva and CSF (2); saliva and nuchal skin (1); saliva, nuchal skin and CSF (1)]. PCR (on single or multiple samples) could therefore confirm the diagnosis in 15 (27.2%) of the 55 laboratory confirmed cases. Of these, PCR alone could confirm the diagnosis in 10 (18.1%) patients.

Of the 15 patients who were positive by PCR on single or multiple samples, 5 had received no prior vaccination and 3 had received partial/complete vaccination; history of vaccination was not known in 7 patients.

### RFFIT

Ante-mortem diagnosis of rabies could be done by RFFIT (in CSF and/or serum) in 45 (81.8%) of the 55 laboratory confirmed cases. Of these 45 cases, RFFIT (in CSF and/or serum) alone could confirm the diagnosis in 40 (72.7%) cases, while the rest of the 5 cases were also positive by PCR (in a single or multiple samples) (Table 1).

### CSF RFFIT

124 CSF samples were received for RFFIT from 100 patients. A single CSF sample was received from 79 patients, 2 CSF samples each from 20 patients and more than 2 (five samples) from one patient. RVNA was detected in CSF samples of 41/100 (41%) patients tested, which contributed to the ante-mortem diagnosis in 74.5% of the 55 laboratory confirmed cases. Titres ranged from 0.1 to 240 IU/ml (mean 32.48 IU/ml). Of these 41 patients positive for RVNA, a single CSF sample was positive in 24 patients, two consecutive CSF samples in 16 patients and more than 2 (5 samples) were positive in one patient.

Of the 41 patients positive for CSF RFFIT, 34 (82.9%) had received prior vaccination (partial or complete), one (2.4%) had not received any prior vaccination and vaccination status was unknown in 6 (14.6%) patients. 17 patients were also positive by serum RFFIT and 3 patients were PCR positive [saliva and CSF positive (2 cases); nuchal skin positive (1 case)]. Therefore, CSF RFFIT alone could confirm a diagnosis of rabies in 21 patients (i.e 38% of the 55 laboratory confirmed cases)

### Serum RFFIT

A total of 111 sera were received for RFFIT from 84 patients. A single serum sample was received from 63 patients, paired sera from 19 patients and more than 2 samples from 2 patients each. RFFIT was positive in sera of 22/84 (26.1%) patients tested. Titres ranged from 0.937 to >960 IU/ml (mean 140 IU/ml).

Of these 22 patients, 5 patients did not receive any prior vaccination (a single serum sample was received from 2 and paired sera were received from 3 patients each), 16 patients had received partial or complete vaccination (paired sera were received from 14 and >2 sera were received from 2 patients each), vaccination status was unknown in 1 patient (paired sera received).

Of the 22 patients who were positive by serum RFFIT, 17 patients were also positive for CSF RFFIT, 2 patients were positive by PCR (in single /multiple samples) and 1 patient was positive by both CSF RFFIT and PCR. Therefore, serum RFFIT alone could confirm a diagnosis of rabies in 2 patients.

### Laboratory confirmed cases (ante-mortem; n = 55)

The age of patients ranged from 1 to 76 years (mean 24.08 years) and 32/55 (58.1%) were male. Children up to 12 years of age accounted for 26/55 (47.2%) of the cases. The presenting clinical features were known in 49/55 laboratory confirmed cases. Based on the manifestations in the acute neurological phase the cases were broadly classified into three clinical forms: encephalitic (agitation, altered mental state, hydrophobia, dysphagia, hypersalivation, signs of autonomic dysfunction), paralytic (muscle weakness, flaccid paralysis) or atypical (not conforming to either encephalitic or paralytic) rabies [15,16]. Based on the clinical profile, 24 cases (49%), 17 cases (34.7%) and 8 cases (16.3%) were classified as paralytic, encephalitic and atypical cases, respectively (S1 Table).

Details on post-exposure prophylaxis (PEP) were available in 43/55 (78.1%) cases; 36/55 (65.4%) cases received vaccination, 7/55 (18.1%) did not receive any vaccination and vaccination status was unavailable in 12 (21.5%) cases. Of the 36 cases who received vaccination, 25 (69.4%) received a complete course of vaccination and 11(30.5%) received partial vaccination. Details on rabies immunoglobulin (RIG) received were available in 37/55 (67.2%) cases. 10/55 (18.1%) received equine or human RIG, 27/55 (49%) did not receive RIG and in 18 (32.7%) cases details were unavailable. The incubation period could be ascertained in 42 cases and ranged from 12 to 90 days (mean 34.6 days). Duration of survival and outcome was known in 39 (70.9%) of the 55 laboratory confirmed cases. Post-mortem brain tissue was not received for testing from any of the 55 cases where confirmation of rabies was done on ante-mortem samples.

In 46/55 laboratory confirmed cases, where details of sample collection were available, the day of collection of samples post-onset of symptoms ranged from 1 day to 64 days (mean 11.06 days). The time from collection of samples to communication of results to the treating physician (including time for transportation of samples to the laboratory) ranged from <1–5 days; mean 3.08 days [1.28 days (range <1–3 days) for PCR results and 3.8 days (range 2–5 days) for RFFIT results].

PCR gave the earliest positive result in 14/46 (30.4%) laboratory confirmed cases and was the only ante-mortem test positive for rabies in 10/46 (21.7%) cases. 7/46 (15.2%) patients were confirmed positive for rabies up to 3 days (post-onset of symptoms); the earliest positive sample was at 1-day (in 1 patient) followed by 2 days (1 patient) and 3 days (5 cases) post-onset of symptoms, respectively. CSF RFFIT was the earliest positive diagnostic test result in 36/46 (78.2%) laboratory confirmed cases and the only ante-mortem test that was positive for rabies in 21/55 (38%) (4 cases were also positive by PCR on samples collected on same day as that of CSF for RFFIT testing). CSF RFFIT was positive by 7 days (post-onset of symptoms) in 12 patients and the earliest positive sample was at 4 days post-onset of symptoms (in 4 patients).

Serum RFFIT was the only test positive in 2 patients; both these patients had received no prior vaccination. Their samples had been collected on day 8 and 13 (post-onset of symptoms) respectively.

**Fig 1:**
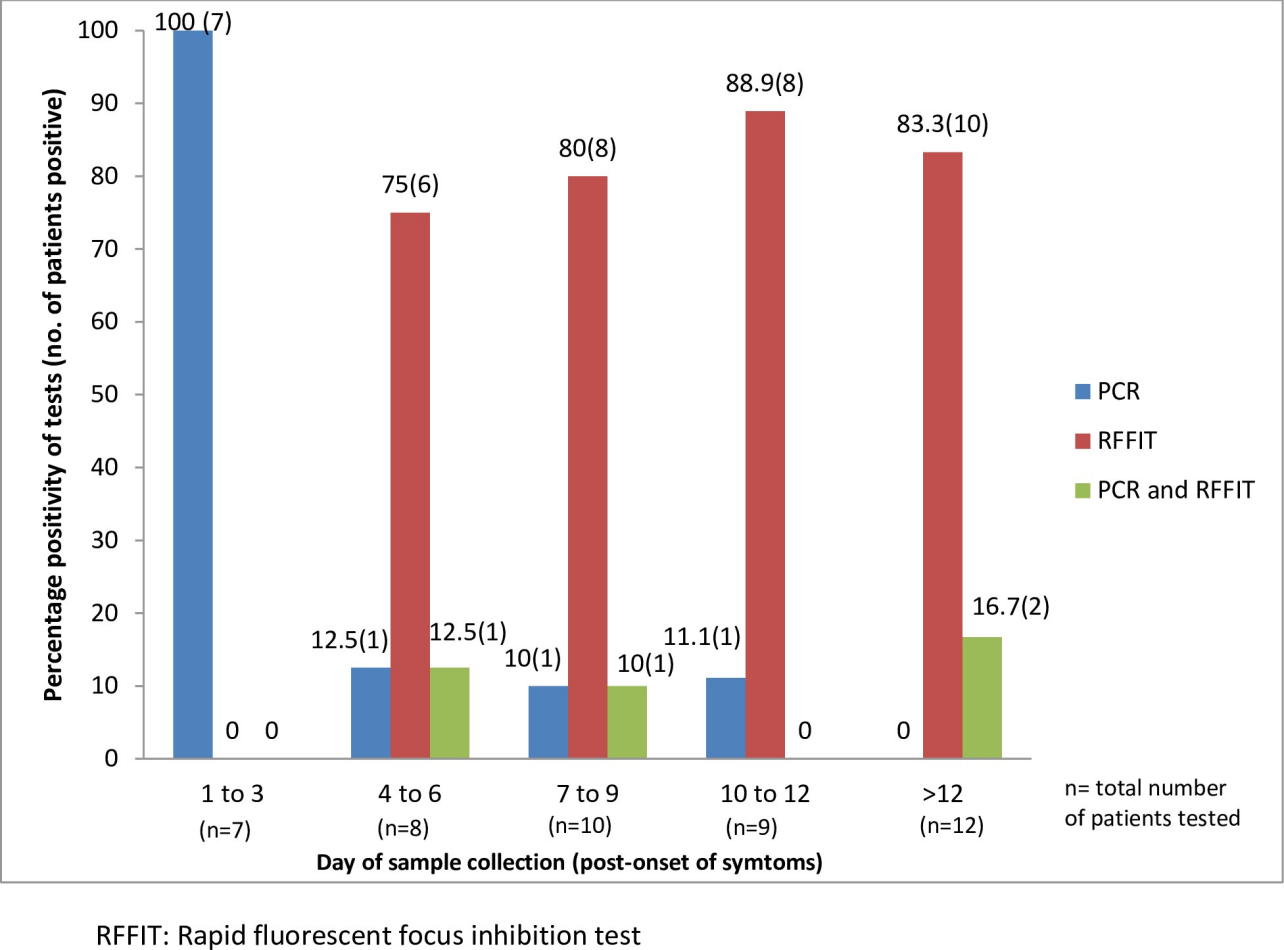
Day of sample collection (post-onset of symptoms) and test positivity in laboratory confirmed cases (PCR and CSF RFFIT).

Fig 1 depicts the day of sample collection (post-onset of symptoms, which ranged from 1 day to 64 days) and test positivity in laboratory confirmed cases. PCR positivity was 100% when the sample was collected within 3 days of onset of symptoms and RFFIT positivity in CSF was 100% when samples were collected after 12 days of onset of symptoms. The correlation of CSF RFFIT titres with day of sample collection (post-onset of symptoms) is presented in Table 2. A positive significant moderate correlation (ρ = 0.603, p = 0.001) between the day of sample collection (post-onset of symptoms) and the CSF RFFIT titres was observed. Results of Kruskal Wallis test showed that there is significant difference between the titres in each group (A, B, C). Multiple comparisons carried out using the Dunn’s post-hoc test depicted a significant difference in the average titres between groups A and B (p = 0.014) and groups A and C (p = 0.001) indicative of higher CSF RFFIT titres being obtained when sample is collected beyond one-week post-onset of symptoms.

**Table 2:** Correlation of CSF RFFIT titres with day of sample collection (post-onset of symptoms).

Day of sample collection	Number of samples	Median (Q3-Q1) Titres in IU/ml	Range (Max-Min) Titres in IU/ml	χ^2^	p-value	Multiple comparison
1–7 (A)	16	0.468 (6.56–0.14)	30–0.1	13.82	0.001	A vs B (p = 0.014)
8–14 (B)	20	11.25 (60–2.34)	120–0.1			A vs C (p = 0.001)
>14 (C)	14	22.5 (60–6.56)	240–1.875			

These 39 laboratory confirmed cases (where the day of CSF sample collection post-onset of symptoms was known) included 37 patients (48 CSF samples) who were positive by CSF RFFIT and 2 patients (2 CSF samples) who were positive by PCR, but negative by CSF RFFIT.

**Fig 2:**
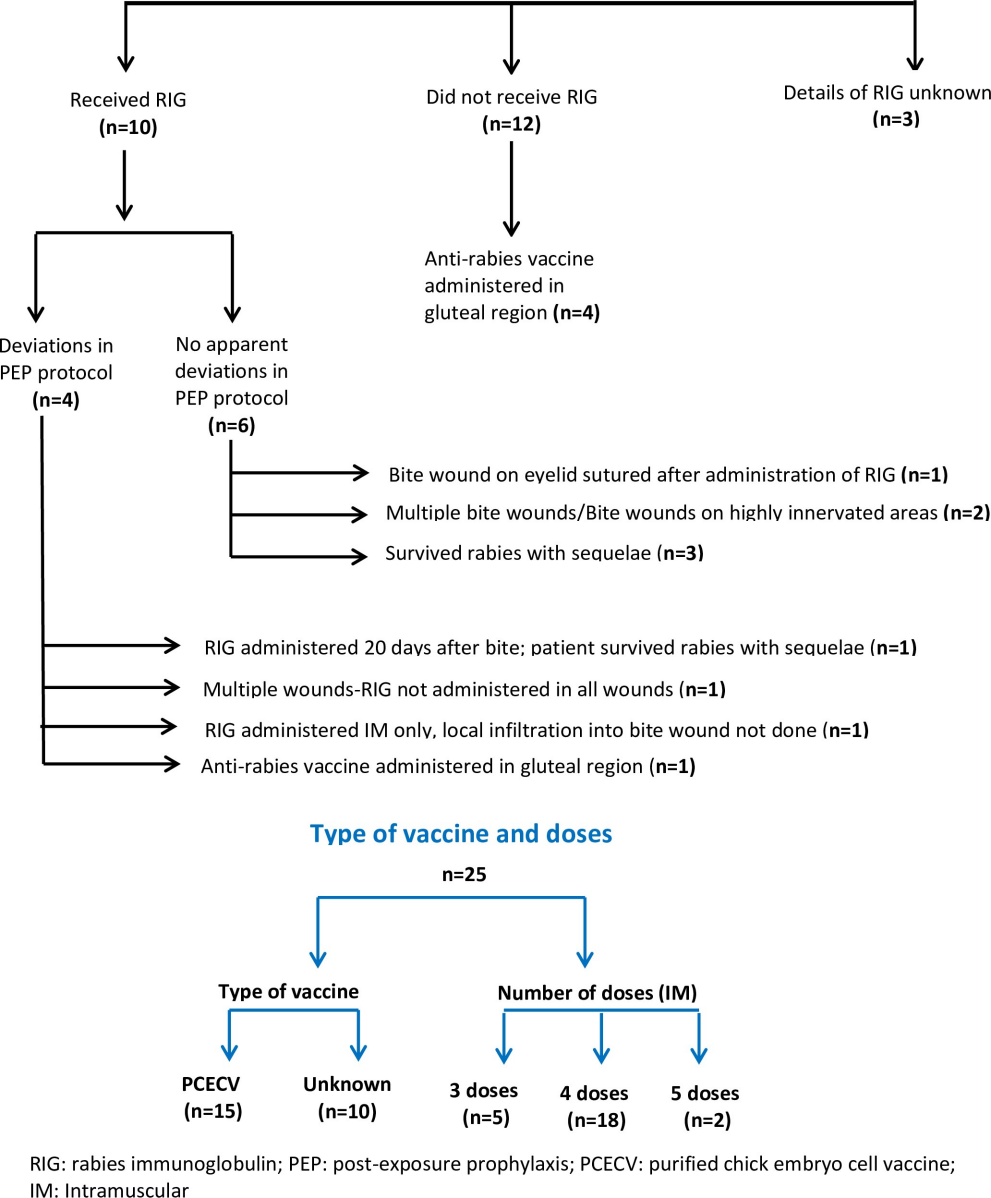
Details of post-exposure prophylaxis in patients with laboratory confirmed rabies who had received complete anti-rabies vaccination prior to symptom onset (n = 25).

**Fig 3:**
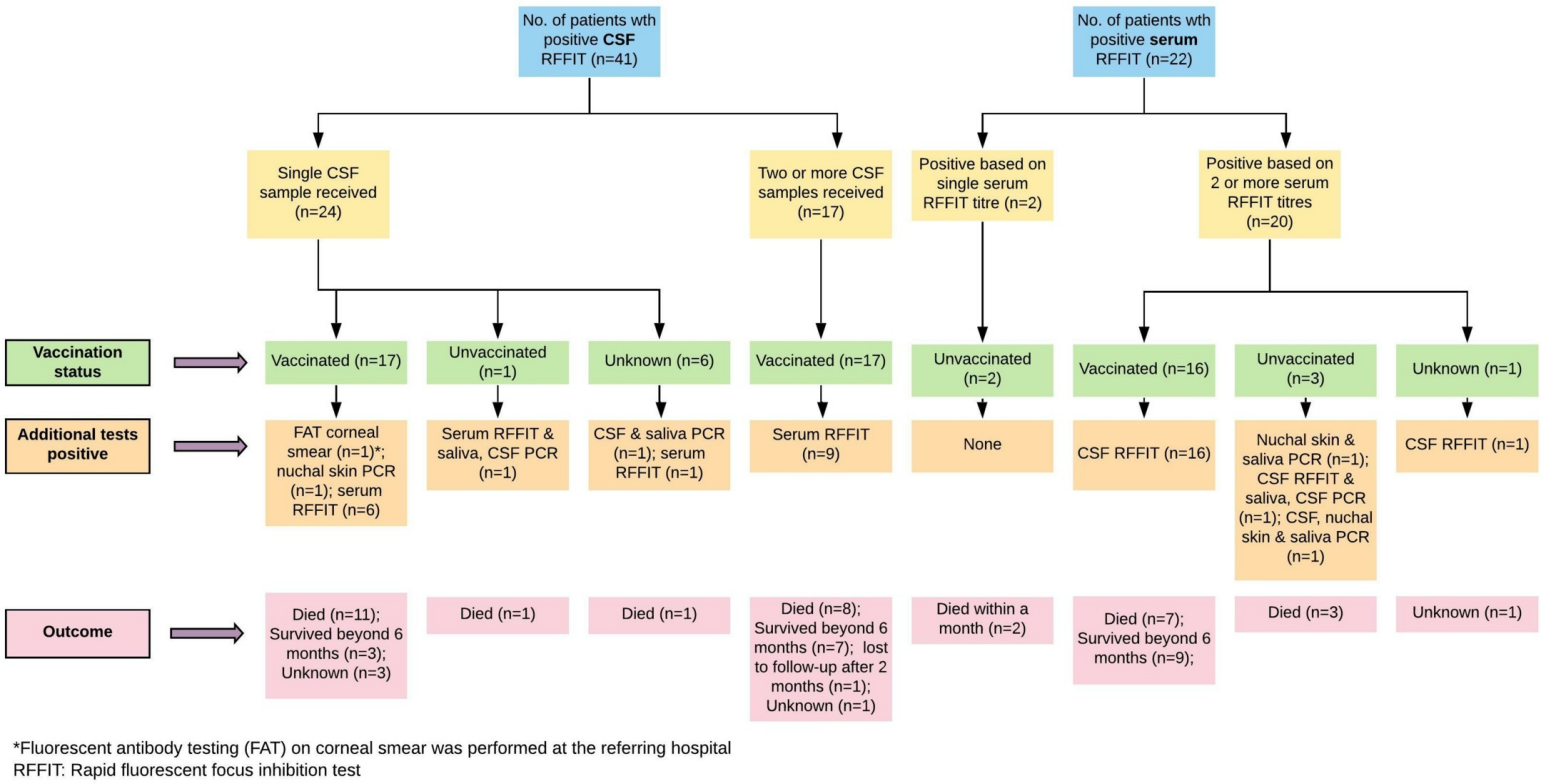
Details of prior vaccination status, number of samples tested, other test results and outcomes in patients who were positive by RFFIT.

Table 3 shows the duration of survival and laboratory test(s) positive at initial diagnosis. Table 4 highlights the association between prior vaccination status and laboratory test positivity. 34/36 (94.4%) vaccinated patients (partial or complete vaccination) were positive by RFFIT (in CSF and/or serum). However, the association between vaccination status and the result of RFFIT was not statistically significant (p = 0.118). In contrast, a significant association was observed between the vaccination status and result of PCR (p = 0.002) where 33/36 (91.7%) vaccinated patients had a negative PCR. In addition, the low probability of a PCR being positive in vaccinated patients as compared to unvaccinated patients is also highlighted by the unadjusted odds ratio (OR = 0.04). Fig 2 shows the PEP details of patients with laboratory confirmed rabies who had received complete anti-rabies vaccination prior to symptom onset. Fig 3 provides details of prior vaccination status, number of samples tested, other test results and outcomes in patients who were positive by RFFIT.

**Table 3:** Duration of survival and laboratory test(s) positive at initial diagnosis (n = 39).

Duration of survival	No of patients (%)	Positive by PCR alone	Positive by RFFIT (CSF/serum) alone	Positive both by RFFIT (CSF/serum) and PCR
< 7 days	4 (10.2%)	4 (100%)	-	-
7–15 days	4(10.2%)	-	2 (50%)	2 (50%)
>15 days- 1 month	8(20.5%)	-	8 (100%)	-
>1 month-3 months	7*(17.9%)	-	5 (71.42%)	2 (28.57)
>3 month-6 months	6^#^(15.3%)	-	6 (100%)	-
>6 months	10^ (25.6%)	-	9 (90%)	1 (10%)

*1 patient lost to follow-up after 2 months.

# 3 patients lost to follow-up after 6 months.

^2 patients died after 6 and 8 months of survival, respectively. 2 cases were lost to follow-up after 7 and 9 months of survival, respectively. 6 patients are still surviving as of June 30^th^ 2018. All survivors (except 2 cases) had/continue to have poor functional outcomes.

5 patients who survived received no intensive care; 3 cases had atypical presentation and 2 had paralytic rabies. 2 patients each survived for 1–3 months and 3–6 months respectively; One survived for >6 months, and has recovered with good functional outcome.

**Table 4:** Association between prior vaccination status and laboratory test positivity.

Test done	Result	Received partial/ complete vaccination		P value	Odd Ratio
		Yes	No		
**PCR**	Positive	3 (8.3%)	5 (71.4%)	0.001	0.04
	Negative	33 (91.7%)	2 (28.6%)		
**RFFIT**	Positive	34 (94.4%)	5 (71.4%)	0.118	
	Negative	2 (5.6%)	2 (28.6%)		

### Laboratory confirmed cases (post-mortem; n = 6)

Post-mortem brain tissue was received from 6 patients. All brain tissues were positive for rabies by FAT and PCR. No ante-mortem clinical samples were received from any of these six patients.

## Discussion

Early laboratory confirmation of rabies can help avoid unnecessary costs and aid in prognostication, decisions regarding treatment or palliation, infection control to limit exposures in healthcare settings, public health measures to investigate the source of infection and provide risk assessments of contacts with potential exposures, and case closure and grief counseling of family members [17,18].

### Diagnostic utility of RFFIT

The RFFIT is considered the gold standard assay and has been used to estimate the post-vaccination titres of RVNA for several years. However, the diagnostic utility of antibody detection in CSF/serum in rabies is usually underestimated/ undermined owing to the late seroconversion and short survival in infected patients. Few studies have highlighted the diagnostic utility of antibody estimation in clinically suspected human rabies cases [5, 19].

As evident from the results of this study, detection of antibodies in CSF and/or serum samples was found to be a valuable ante-mortem diagnostic tool in clinically suspected cases of human rabies. Ante-mortem diagnosis of rabies could be achieved by RFFIT (in CSF and/or serum) in 45 (34.6%) of the 130 clinically suspected cases, accounting for 81.8% of the total 55 laboratory confirmed cases. Of these 45 cases, RFFIT (in CSF and/or serum) alone could confirm the diagnosis in 40 (72.7%) cases, while the rest of the 5 cases were also positive by PCR (in a single or multiple samples).

CSF RFFIT was the earliest positive diagnostic test result in 36/46 (78.2%) laboratory confirmed cases and the only ante-mortem test that was positive for rabies in 21/55 (38%). CSF RFFIT was positive by 7 days post-onset of symptoms in 12 (26%) patients and the earliest positive sample was at 4 days post-onset of symptoms in 4 (8.6%) patients. Serum RFFIT was the only test positive in 2 patients; both these patients had received no prior vaccination. Their samples had been collected on day 8 and 13 (post-onset of symptoms) respectively.

Ante-mortem laboratory confirmation of rabies infection could be achieved in 18 of the 20 clinically suspected and previously unvaccinated cases of human rabies diagnosed in the United States from 1980 to 1996. Antibody to RABV was detected in the sera of 10 (50%) patients; it was the earliest positive diagnostic test result in 7 (35%) patients and the only ante-mortem test that was positive for rabies in 3 (15%) cases. Antibody in CSF was detected in 2 (15.3%) of the 13 patients for whom CSF was submitted for ante-mortem diagnosis of rabies [19]. Neutralizing antibodies in CSF were detected in 20 (22.4%) of 89 clinically suspected cases of rabies in an earlier study from India [5]. In another review of the laboratory diagnostic testing for all reported human rabies cases in the United States between 1960 and 2010, antibodies in serum and CSF were detected in 33/48 (69%) and 17/40 (43%) unvaccinated patients, respectively. Antibodies were detected in serum and CSF at a median of 10 (range 4–21) and 14 (range 5–28) days respectively following the onset of symptoms; the earliest detection of rabies specific antibodies after illness onset was 4 days in serum and 5 days in CSF [18]. In contrast, antibodies could be detected by RFFIT in the sera of only 6 (19.3%) of the 31 non-vaccinated Thai patients with canine mediated rabies tested within 1–26 days after onset of disease. CSF antibodies could not be demonstrated in the CSF of any of the 27 patients [20], which is unusual and difficult to explain regardless of the vaccination status.

Serum antibody to rabies virus is not present until several days after the onset of clinical signs and appears even later in CSF. Therefore, antibody detection is of limited value for diagnostic confirmation early in the course of illness [19, 21]. However, the sensitivity of antibody detection in CSF and serum increases beyond a week and is >90% after 2 weeks of illness [3]. Therefore, in patients where the survival is prolonged beyond one to two weeks [22–24] antibody detection has a vital diagnostic role [5].

As observed in this study (Fig 1), the sensitivity of CSF RFFIT increases with the day of sample collection (post-onset of symptoms) and was found to be 100% when samples were collected after 12 days of illness. We also observed a significant correlation between the day of sample collection and the CSF RFFIT titres (Table 2). The median titres of RFFIT increased from 0.46 IU/ml (<7 days) to 22.5 IU/ml (>14 days), indicative of significantly higher CSF RFFIT titres being obtained when sample is collected beyond one-week post-onset of symptoms.

A majority (65.3%) of the cases in our study had paralytic or atypical features and a protracted course of illness. Paralytic rabies is more frequently observed in patients who are partially vaccinated, possibly resulting from an immunopathologic attack on virus infected cells in the brain and spinal cord [25]. The duration of survival of patients ranged between 3 days to > 1 year and 41% of the patients survived beyond 3 months. This may represent a referral bias, since most samples received for diagnostic confirmation in our laboratory are from paralytic/atypical cases which have relatively longer survival periods [22], may mimic other clinical conditions [3, 17], and pose a diagnostic dilemma to treating physicians, unlike in patients with classical encephalitis (hydrophobia) where laboratory assistance may not always be required for confirmation of diagnosis. Nevertheless, this study highlights the changing clinical profile of rabies and the increasing number of paralytic/atypical cases being seen, hitherto considered uncommon, about which physicians in rabies endemic countries need to be aware of.

Another factor which may contribute to the low sensitivity of antigen or nucleic acid detection (e.g PCR) and increased sensitivity of antibody detection is the vaccination status of the individual prior to development of symptoms. Though the association between vaccination status and the result of RFFIT was not found to be statistically significant (p = 0.118), a majority of patients (94.4%) who had received prior vaccination (partial or complete) were positive by RFFIT (in CSF and/or serum). In contrast, most of the vaccinated patients (91.7%) tested had a negative PCR. A significant association was observed between the vaccination status and result of PCR (p = 0.002), reinforced by the unadjusted odds ratio (OR = 0.04) indicating a lesser likelihood of PCR being positive in vaccinated patients as compared to unvaccinated patients (Table 4). An inverse correlation has been reported between the presence of neutralizing antibodies and detection of viral RNA in clinical samples [4, 26]. Patients who have received prior vaccination but develop rabies (usually due to lack of RIG administration and/or inadequate vaccine doses) may rapidly develop high concentrations of serum and CSF-neutralizing antibodies, which can explain the low sensitivity of PCR on various clinical samples obtained ante-mortem in our study.

Results of serological assays have to be interpreted with caution when the test is performed on a single CSF or serum sample, especially when the history of vaccination is unavailable or unreliable. Recent intravenous immune globulin (IVIG) administration is known to confound serologic diagnosis of rabies [27]. Though the presence of specific antibodies in CSF is considered diagnostic of rabies irrespective of vaccination status [28], breach in blood brain barrier due to any other infectious or non-infectious etiology or a traumatic tap can give false-positive results in vaccinated individuals. Demonstrating a fourfold or higher rise in titres in paired CSF and/or serum samples drawn 7–10 days apart can confirm a diagnosis of rabies [29], since a rise in titres can occur only due to the natural disease process and not as a result of prior vaccination, usually received weeks to months before onset of symptoms. To this end, additional details for patients who were positive by RFFIT are provided (in Fig 3) for validation of the RFFIT results in this study. Briefly, of the 41 patients positive by CSF RFFIT, 34 (82.9%) had received prior vaccination. Of these 34 patients, 17 (50%) patients were positive for RFFIT in two or more samples (with a four-fold or greater rise in titres). In the rest of the 17 cases who were vaccinated and positive by RFFIT in a single CSF sample, 8 (47%) patients were also positive by other tests-nuchal skin PCR positive (1), RFFIT positive in paired sera (6) and FAT corneal smear (1). Therefore in 25/34 (73.5%) patients the CSF RFFIT positivity has been substantiated by additional evidence. Similarly, of the 22 patients who were positive by serum RFFIT, 17 (77.2%) had been vaccinated. Paired sera were tested in all these 17 cases which showed four-fold or greater rise in titres. All these 17 cases were also positive by CSF RFFIT. All patients whose samples were sent had a history of exposure and a clinical illness compatible with rabies, where most of the other differential diagnoses had been ruled out by the treating physicians.

### Prognostic utility of neutralizing antibody detection

Individuals who develop neutralizing antibodies in the serum or CSF early during the disease course are considered potentially favourable candidates for aggressive management [30]. In patients being treated with the experimental protocols, serology provides a useful tool to monitor the titres of CSF neutralizing antibodies and thus possible clearance of the RABV from the central nervous system [26]. Survival has been associated with an early appearance of CSF-neutralizing antibody titers in the range of 1.0–10 IU/ml [17]. Notably, most documented survivors of rabies, with or without treatment, had a vigorous early immune response and were diagnosed solely by the presence of neutralizing antibodies in the CSF and/or serum, but no demonstrable viral antigen or nucleic acid [1, 31].

In our study, the duration of survival and outcome of illness was known in 39 (70.9%) of the 55 laboratory confirmed cases. 16 of the 39 (41%) patients survived beyond 3 months and RFFIT (in CSF and/or serum) was the only test positive at initial diagnosis in 15 (93.7%) of them, while 1 (6.2%) patient was positive by both RFFIT and PCR. In contrast, all 4 patients (100%) who were positive by PCR only at initial diagnosis died within a week. Patients who were positive by RFFIT alone at initial diagnosis had longer survival (albeit with moderate to severe neurological sequelae) than patients who were positive by PCR alone or patients who were positive both by RFFIT and PCR (Table 3).

All the patients in this study who had longer survival were managed with supportive measures with or without intensive care. The longer duration of survival can be attributed to vaccination prior to onset of illness (partial or complete), increased awareness about rabies, ante-mortem laboratory confirmation and better access to critical care facilities in India in recent years. However, tragically, as observed in this study too, most of the reported human survivors in India are left with poor functional outcomes, with serious long-term repercussions for care-givers [24].

These cases re-emphasize the importance of timely, appropriate and adequate PEP to prevent rabies and draws attention to the serious repercussions of incomplete PEP or deviations as observed in this study. Though recovery from rabies is consistently associated with the presence of neutralising antibodies in the blood and CSF, family members of previously vaccinated patients diagnosed with rabies must be made aware that although intensive care can prolong life, the patient is most likely to survive with multiple disabilities. These cases also underscore the urgent need for formulation of national guidelines for management of rabies cases in rabies endemic countries and the imperative of humane palliative care to minimize the suffering of those dying of this agonizing disease [32].

Current medical outcomes, however dismal, do not predict future medical outcomes, as evident by the commendable strides in medicine over the last few decades [24]. Though the number of current survivors is small, an increasing number of survivors are being reported from India in recent years and probabilistically, better outcomes will follow. In fact, 10 patients in this study (7 cases published [33]) have survived beyond 6 months, two of them with good functional outcomes (Table 3).

There are several challenges associated with neutralization assays like the RFFIT- cumbersome testing procedure and extended turn-around-time (2–3 days), requirement of cell culture facility, fluorescent microscope and biosafety measures. Therefore, in laboratories which do not have facilitates to perform RFFIT, an adequately validated ELISA for antibody detection against RABV G protein can be used. An ELISA is an easier, faster, cheaper and safer (use for live virus not required unlike RFFIT) alternative and been shown to correlate well with virus neutralization assays like RFFIT [34, 35]. Rapid detection of antibodies (IgG and IgM) to other viral antigens, (e.g. nucleoprotein) may also be useful, as they may appear before neutralizing antibodies [1]. Rabies-specific IgM is known to appear in serum earlier than IgG or total neutralizing antibody and therefore it was suggested that detection of rabies specific IgM in the CSF of infected patients may be a rapid method of early diagnosis [36]. However IgM detection in CSF and serum was found to be of limited utility in diagnosis of rabies in the first week of illness [37].

This study highlights a critical observation with serious public health implications-an increasing number of vaccinated individuals continue to develop and die of rabies and a few survive with serious disabilities (Fig 2). There was no apparent deviation in PEP protocols used in six laboratory confirmed rabies cases, who had received both vaccination and RIG. Three of these patients survived rabies with sequelae. In the rest of the three patients who died, these PEP failures, which are extremely rare [38], can be attributed to multiple bite wounds and/or bites on highly innervated areas; one also had a bite wound on the eyelid which was sutured. In another recent study published from a large public infectious diseases hospital in Delhi, India, 783 cases of rabies, (hydrophobia) were admitted in the last 10 years. Notably, 246 (32%) of them had received ARV; only 22 (9%) of these 246 cases received RIG also, while the rest of them i.e 224 (91%) did not receive RIG [39]. As amply evident, these PEP failures can be attributed to serious omissions/deviations in the PEP protocol used. This can adversely impact the rabies control initiatives in India which accounts for a high disease burden, and necessitates urgent efforts towards increasing awareness about rabies in the public and amongst health professionals.

Our study has some limitations. The details of ARV included information about vaccination only as a part of the recent PEP for all patients. Reliable data on any other past history of rabies pre-exposure or post-exposure prophylactic vaccination could not be obtained. Since the diagnostic tests for the clinically suspected cases were performed as requested by the referring physicians, not all tests were performed for all patients. Moreover, since our laboratory receives samples from various hospitals across the country, serial sampling and testing of multiple clinical samples which could have increased the overall sensitivity of ante-mortem diagnosis, was not feasible for all patients. Post-mortem brain tissue was received only from six suspected cases and in none of the 130 patients where ante-mortem clinical samples were sent, re-iterating the challenges in post-mortem confirmation of human rabies. Therefore, post-mortem confirmation by the ‘gold standard’ FAT on brain tissue could not be done on any of these cases. Once a diagnosis of rabies is made, family members often opt to take the patient home to avoid health care costs. Therefore, the outcome of several patients was not known/documented. Laboratory tests for rabies were negative on ante-mortem clinical samples in 75/130 (57.6%) patients with clinically suspected rabies. Rabies could not be ruled out in these patients-a well-recognized limitation of ante-mortem diagnostic testing.

In conclusion, this study highlights the challenges of ante-mortem rabies diagnosis and re-iterates that the best approach for maximizing the sensitivity and accuracy of ante-mortem diagnosis of rabies is through testing of multiple/serial samples using multiple modalities. Despite the challenges in interpretation, detection of specific antibodies in the CSF and/or serum is a valuable adjunctive/confirmatory ante-mortem diagnostic tool in human rabies, especially when other tests may be non-contributory, particularly in the context of a changing clinical profile of rabies (vaccinated cases with protracted course of illness) in rabies endemic countries in recent years- which may not conform to the clinical and laboratory profile of rabies described several years ago, that the scientific community even today largely identifies with. Early presence of CSF neutralizing antibodies in previously vaccinated/unvaccinated rabies cases can also potentially be used to help guide decisions regarding intensive /supportive /palliative care in patients along with several other factors [30] and as a prognostic marker to predict outcomes, albeit with limited value.

## Supporting information

S1 Table Presenting clinical features of laboratory confirmed cases (n = 49). (DOCX)
