# Supplementary material for: Utility of rabies neutralizing antibody detection in cerebrospinal fluid and serum for ante-mortem diagnosis of human rabies
Source: PLoS Negl Trop Dis. 2019 Jan 29;13(1):e0007128. doi: 10.1371/journal.pntd.0007128 (PMC6368332; doi:10.1371/journal.pntd.0007128)
Supplement: S1 Table — (DOCX) [file pntd.0007128.s001.docx]

**S1 Table: Presenting clinical features of laboratory confirmed cases (n=49)**

| **S. No** | **Age in years** | **Sex** | **Clinical features** | **Clinical Form** |
| --- | --- | --- | --- | --- |
| 1* | 10 | M | fever, lethargy, altered sensorium | Encephalitic |
| 2 | 18 | M | fever, drowsiness, delirium, bipyramidal weakness (upper limb more than lower), bilateral ptosis, respiratory distress requiring ventilator | Paralytic |
| 3 | 42 | F | fever with chills and irrelevant talk | Atypical |
| 4 | 29 | M | fever with abdominal pain, episodes of panting, hydrophobia, aerophobia | Encephalitic |
| 5 | 32 | M | fever, right lower limb pain, hydrophobia and aerophobia, irritability and altered sensorium, multiple episodes of vomiting and autonomic hyperactivity | Encephalitic |
| 6* | 10 | M | fever, bilateral lower limb weakness followed by upper limbs involvement | Paralytic |
| 7 | 55 | M | lower limb weakness, followed by ascending paralysis, respiratory muscles involved, requiring ventilatory support | Paralytic |
| 8 | 7 | M | Fever, weakness of lower limb, ascending paralysis | Paralytic |
| 9 | 3 | F | fever followed by decreased levels of consciousness leading to stuporous state | Encephalitic |
| 10* | 5 | M | fever, altered sensorium, drowsiness, neck stiffness and hyperreflexia | Encephalitic |
| 11 | 16 | F | fever, difficulty in swallowing, increased salivation, ataxia, poor neck holding | Atypical |
| 12 | 60 | F | fever, hearing loss, weakness and numbness of both lower limbs, difficulty in walking | Paralytic |
| 13 | 10 | F | hydrophobia, behavioural changes, altered sensorium, drowsiness, fever, neck stiffness, hyperreflexia | Encephalitic |
| 14 | 18 | M | fever, hydrophobia and aerophobia | Encephalitic |
| 15* | 18 | F | fever, projectile vomiting, unsteady gait, ataxia | Atypical |
| 16 | 42 | F | progressive ascending bilateral lower limb weakness, difficulty in swallowing, altered sensorium | Paralytic |
| 17 | 29 | F | Numbness at site of bite, aerophobia, hydrophobia, agitation | Encephalitic |
| 18 | 76 | M | right upper limb weakness, difficulty in walking, slurring of speech | Paralytic |
| 19 | 64 | F | left lower limb flaccid weakness followed by right lower limb, difficulty in swallowing, rapidly progressive upper limb weakness, difficulty in breathing, urinary retention | Paralytic |
| 20 | 10 | M | fever, altered sensorium, flaccid paralysis of both lower limbs, progressive worsening of GCS (Glasgow Coma Scale) | Paralytic |
| 21 | 3 | M | fever, altered sensorium, reduced oral intake, paradoxical breathing | Encephalitic |
| 22 | 12 | M | fever, flaccid paralysis of both lower limbs | Paralytic |
| 23 | 10 | F | fever, altered sensorium, drowsiness, neck stiffness, extensor plantars, hyper-reflexia | Encephalitic |
| 24 | 1.5 | F | screaming and transient startling episodes, vomiting, fever, lethargy, unable to walk, loss of head control and upper limb weakness, poor swallowing | Atypical |
| 25 | 13 | M | fever, breathlessness, paralysis, ?aerophobia | Paralytic |
| 26 | 10 | M | acute febrile encephalopathy | Encephalitic |
| 27 | 4 | F | fever, vomiting, dysphagia, rapidly progressing lower limb weakness | Paralytic |
| 28 | 5 | F | fever, gradually stopped talking | Atypical |
| 29 | 42 | M | ? GBS (Guillain Barre syndrome); details not mentioned | Paralytic |
| 30 | 45 | M | weakness of all limbs, respiratory paralysis requiring ventilator | Paralytic |
| 31 | 12 | M | headache, anxiety, hallucinations | Atypical |
| 32 | 1 | F | fever, lower limb weakness | Paralytic |
| 33* | 5 | F | fever, vomiting, drowsiness, altered sensorium, neck rigidity | Encephalitic |
| 34 | 45 | M | fever, extreme agitation, flaccid paralysis | Paralytic |
| 35 | 16 | M | Fever, altered sensorium, lower limb weakness | Paralytic |
| 36 | 4 | F | fever, seizures, vomiting, altered sensorium | Encephalitic |
| 37* | 9 | M | fever, neck pain, pain in upper limb, agitation, somnolence, quadriparesis, loss of speech | Paralytic |
| 38 | 26 | M | fever, body ache, ear discomfort, diplopia, difficulty walking, behavioural changes- restlessness, excessive and irrelevant talking | Atypical |
| 39* | 4 | M | fever altered sensorium (drowsiness), oculogyric crisis, quadriparesis | Paralytic |
| 40 | 55 | M | tingling in limbs and back, fever with chills and hydrophobia, agitated and aggressive | Encephalitic |
| 41 | 4 | F | fever, lower limb paresis | Paralytic |
| 42* | 5 | F | fever with chills, drowsiness, projectile vomiting | Encephalitic |
| 43 | 25 | M | weakness in lower limbs, drowsiness, right facial palsy | Paralytic |
| 44 | 3 | M | flaccid weakness of all limbs with ventilatory support | Paralytic |
| 45* | 3 | F | excessive sleep, lethargy, drowsiness, decorticated posture, unable to walk, no response to verbal commands | Paralytic |
| 46 | 8 | M | hydrophobia, hyperactive behaviour, fever and hallucinations | Encephalitic |
| 47* | 26 | M | fever, altered behaviour, weakness of lower limbs | Paralytic |
| 48 | 56 | M | restlessness, monoplegia (right upper limb) | Atypical |
| 49 | 54 | F | altered sensorium, hydrophobia, aerophobia, difficulty in swallowing | Encephalitic |
|  |  |  |  |  |

*Survived beyond 6 months: Case numbers 1, 33, 37, 39, 42, 45, 47 (published; reference 33)
